# Network modeling of problematic social media use components in college student social media users

**DOI:** 10.3389/fpsyt.2024.1386845

**Published:** 2025-01-13

**Authors:** Jianyong Chen, Ting Su, Junqiang Dong, Yuzhi Li, Ju Feng, Yingxiu Chen, Gu Liu

**Affiliations:** ^1^ School of Psychology, Zhejiang Normal University, Jinhua, China; ^2^ Intelligent Laboratory of Child and Adolescent Mental Health and Crisis Intervention of Zhejiang Province, Zhejiang Normal University, Jinhua, China; ^3^ Mental Health Education Center, Zhejiang A & F University, Hangzhou, China

**Keywords:** problematic social media use, network analysis, Gaussian graphical model, directed acyclic graph, components model of addiction

## Abstract

**Background:**

While the constitutive features of problematic social media use (PSMU) have been formulated, there has been a lack of studies in the field examining the structure of relationships among PSMU components.

**Method:**

This study employed network analytic methods to investigate the connectivity among PSMU components in a large sample of 1,136 college student social media users (*M*_age_ = 19.69, *SD* = 1.60). Components of PSMU were assessed by the Bergen Social Media Addiction Scale (BSMAS) derived from a components model of addiction. We computed two types of network models, Gaussian graphical models (GGMs) to examine network structure and influential nodes and directed acyclic graphs (DAGs) to identify the probabilistic dependencies among components.

**Result:**

Relapse component consistently emerged as a central node in the GGMs and as a parent node of other components in the DAGs. Relapse and tolerance components exhibited strong mutual connections and were linked to the most vital edges within the networks. Additionally, conflict and mood modification nodes occupied more central positions within the PSMU network for the low-BSMAS-score subgroup compared with the high-BSMAS-score subgroup.

**Conclusion:**

Our findings shed new light on the complex architecture of PSMU and its potential implications for tailored interventions to relieve PSMU.

## 1 Introduction

At present, the use of social media on different platforms (e.g., Facebook, WeChat, TikTok) is an integral part of university students’ everyday life. Social media enable people to make new friends and maintain contacts with existing social network members without geographical or time constraints (1). Upon entering university, parental monitoring and restrictions for Chinese students significantly decrease, providing increased autonomy and independence (2). On the other hand, the new environment introduces pressures related to forming interpersonal relationships and academic competition (3). Consequently, Chinese students may resort to social media to fulfill social needs and cope with stress (4), leading to frequent and extensive engagement with social media. In China, 99.4% of college students use social media daily (4). Among them, 25.1% spend less than 3 hours per day on social media, 52.3% dedicate 4-6 hours daily, and 22.6% use social media for more than 7 hours each day (4). However, frequent social media usage may increase the risk of developing problematic social media use, especially in younger age groups (1). Problematic social media use (PSMU) is defined as excessive and uncontrolled use of social media, which leads to detrimental consequences on the users’ functioning and psychological wellbeing (5). The prevalence estimates of PSMU in Chinese college students range from 28.6% to 44.5% (6, 7). PSMU is associated with a range of negative health consequences including decreased mental well-being (e.g., anxiety and depression) (8), more sleep problems (9), poor academic performance (10), and less healthy social relationships (11) among Chinese college students. Thus, it is imperative to examine the underlying mechanisms of the development and maintenance of PSMU in college students.

To date, the most cited theoretical framework for PSMU may be the components model of addiction (12). It conceptualizes and diagnoses PSMU based on six components: *salience*, involving cognitive, emotional, and behavioral preoccupation with social media usage; *tolerance*, indicating increasing involvement in social media use to obtain a previous effect; *relapse* (or *loss of control*), reflecting unsuccessful attempts to cut down on social media use; *withdrawal*, involving unpleasant physical and emotional experiences when social media use is restricted or stopped; *mood modification*, indicating engagement in social media to achieve a favorable change in emotional states; and *conflict*, encompassing intrapersonal and interpersonal conflicts arising from engagement in social media usage (13, 14).

Building on this theoretical basis, researchers have developed various measurement tools to evaluate problematic use of social media (13) or specific social media platforms (15). Among these, the Bergen Social Media Addiction Scale (BSMAS) (13) is a widely used instrument in the research of PSMU (12, 16). This scale includes six items, each of which assesses one component. Many previous studies using either exploratory or confirmatory factor analysis have identified a single factor solution as the best latent model of the BSMAS’s items in Chinese (17, 18), Korean (19), Bangladeshi (20), and Italian (21) populations, with the six items loading onto a single higher-order latent variable: PSMU. The BSMAS demonstrated adequate internal consistency reliability (17–21) and test-retest reliability (19). The total scores across items were typically used to represent the level of PSMU (17–21). However, the debate on the appropriate BSMAS cut-off score for identifying PSMU remains ongoing (22). In light of this, a recent meta-analysis (22) suggested using the average scores of BSMAS instead of cut-off points, with individuals scoring above the averages considered “positive” for PSMU or at an increased likelihood of it. Studies revealed that college students with a higher total score on BSMAS spent more time on social media (18, 20) and engaged in social media use more frequently (23). However, the use of total scores may obscure the meaningful ongoing developments among the six components of PSMU, as well as hinder clarification of whether some components may be more central to the development of PSMU than others (24).

In recent years, the emerging perspective of network analysis has offered an alternative way to understand and conceptualize disorders. According to the network perspective, disorders are conceptualized as network systems of interacting observable indicators (i.e., scale items) rather than as effects of a latent disorder (25). As such, the emergence of PSMU arises from the interactions among its constituent indicators. Previous network studies have estimated Gaussian graphical models (GGMs) in which the *nodes* represent indicators of PSMU and the *edges* connecting the nodes signify the strength of linkage between pairs of nodes after controlling for all other nodes in the network (24, 26). The presence of edges suggests that two nodes are likely to occur simultaneously. In contrast, the absence of edges implies conditional independence between the two nodes. These network analyses have the potential to clarify the PSMU construct because they enable identification of the most central nodes and edges acting as main pathways of the network (24, 26).

Some previous studies identified items representing relapse and tolerance as the highest central nodes in the PSMU network (24, 26), while items representing salience as a peripheral node (5, 26, 27). However, Fournier et al. (27) and Peng et al. (5) classified items representing tolerance (and salience) as periphery features because these nodes exhibited no associations with psychopathological symptoms (e.g., anxiety and depression) in the network. Of note, neither of the two studies reported associations between PSMU components and engagement in social media (e.g., duration of use), which limits our understanding of the ability of the two components to distinguish across different users and potentially affects the validity of the results. Moreover, unique components may emerge across different severity stages (28). For example, Király et al. (28) discovered that components of mood modification, conflict, salience, and continuing despite harms were more endorsed frequently among internet gamers in less severe stages, while relapse, tolerance, giving up other activities, and deception were only reported in more severe cases. To date, little is known about whether and how core components in PSMU network differ by severity of PSMU.

Recently, many longitudinal network studies have examined the changes of core nodes related to problematic internet use (PIU) (29–31). For instance, Zhao et al. (29) identified “using the internet to escape problems” and “feeling irritable when offline” as central nodes in the comorbidity network of PIU and depression across two waves. Additionally, Huang et al. (30) found that the item describing “continued excessive smartphone use” had the highest centrality in the temporal and contemporaneous networks, and the item representing “relapse” served as a key node in the contemporaneous network. Nevertheless, extant studies have primarily focused on the core component changes in the generalized PIU network or the comorbidity networks of PIU with other related conditions. Consequently, the specific nature of associations between individual components within specific PIU types, such as PSMU, remains largely unexplored.

Bayesian network methods offer a complementary way to delineate components as a system of interacting nodes (32, 33). These methods estimate directed acyclic graphs (DAGs), which encode the conditional independence relationships between the nodes and depict the joint probability distribution of the nodes (32, 33). A DAG possesses arrows representing the direction of probabilistic dependence between connected components, but it prohibits cycles among components (e.g., X→Y→X) (32, 33). While cross-sectional DAGs cannot determine temporal precedence, they can identify *directional dependence relations* in which the presence of a specific node (i.e., a descendant node) is more likely to imply the existence of another node (i.e., a parent node) than vice versa (32, 33). In other words, a parent node may often be present without its descendant, while the presence of the descendant node implies the existence of the parent node. Therefore, DAGs may help to gather information about the potential mutual relationships between components.

This exploratory study employed both regularized (and nonregularized) partial correlation and Bayesian network methods to estimate network structure of PSMU measured by the BSMAS (13). Our investigation contributes to the field since it adds evidence to identify component relationships and influential nodes in the network of PSMU. To test the validity of the BSMAS, we evaluated participants’ problematic short video social media use, social anxiety, and maladaptive cognitions toward social media use. We assumed that there would be significant positive associations among these variables (H1).

To delineate the position and importance of each component, we estimated the centrality of each component in the partial correlation networks, and identified parent nodes in the Bayesian networks (32). Moreover, we examined whether the core components varied based on the severity of PSMU. A drug addiction theory, the impaired response inhibition and salience attribution (iRISA) model (34), proposes that two key neuropsychological functions contribute to the development of core symptoms of addiction: inhibitory control (regulated by the dorsal prefrontal cortex) and salience attribution (regulated by the ventral prefrontal cortex). The iRISA model posits that decreased inhibitory control and increased incentive salience attributed to drugs or drug-related cues leads to the emergence of addiction symptoms like craving, withdrawal, and bingeing (34). Moreover, previous network studies have highlighted relapse as a central feature in the network of PSMU (24, 26). Thus, we hypothesized that the item representing relapse would function as a central and parent node across networks within the entire sample (H2). Building upon existing literature (28), we predicted that components such as mood modification and conflict would hold more central positions in the networks for users in less severe stages (H3).

## 2 Methods

### 2.1 Participants and procedures

Between February and June 2022, a total of 1,296 full-time undergraduates participated in the current study. The participants were recruited from two universities in Zhejiang province of China via posters, flyers, and contacting their instructors. To be eligible to participate, students had to have at least one active social media account and use social media at least once per week for a minimum of one year (35). Social media was defined as “WeChat, TikTok, QQ, and the like” in the instructions to participants. Students who did not use social media or did not want to participate in the study were excluded. Participation was voluntary, and no incentives were provided. One hundred and sixty participants were excluded, as they failed to answer the reading check question correctly (i.e., As a reading check could you please select the answer “I agree strongly”) (*n* = 103) or provided incomplete data (e.g., participants did not complete the items after filling out the demographic variables) (*n* = 57). The final sample consisted of 1,136 participants (63.1% female; age range = 17-23 years, *M*_age_ = 19.69, *SD* = 1.60) (see **Table 1**).

**Table 1:** Entire sample characteristics and associations with level of problematic social media use (PSMU) severity (*N* = 1136).

Variables		Entire sample	Level of PSMU severity		*t*/*χ* ^2^	*P*
			Low	High		
Age (mean ± SD)		19.69 ± 1.60	19.51 ± 1.53	19.86 ± 1.53	3.84	< 0.001
Gender	Female	717 (63.1)	357 (49.8)	360 (50.2)	3.02	0.082
	Male	419 (36.9)	231 (55.1)	188 (44.9)		
Grade	Freshman	451 (39.7)	277 (61.4)	174 (38.6)	31.83	< 0.001
	Sophomore	248 (21.8)	101 (40.7)	147 (59.3)		
	Junior	253 (22.3)	118 (46.6)	135 (53.4)		
	Senior	184 (16.2)	92 (50.0)	92 (50.0)		
Residence	Rural	510 (44.9)	264 (51.8)	246 (48.2)	< 0.01	0.998
	Urban	626 (55.1)	324 (51.8)	302 (48.2)		
Only child	Yes	495 (43.6)	261 (52.7)	234 (47.3)	0.33	0.57
	No	641 (56.4)	327 (51.0)	314 (49.0)		
Family economic status	Poor	141 (12.4)	79 (56.0)	62 (44.0)	1.19	0.55
	Average	963 (84.8)	493 (51.2)	470 (48.8)		
	Well-off	32 (2.8)	16 (50.0)	16 (50.0)		
Social media use duration (mean ± SD)		5.08 ± 1.14	4.79 ± 1.23	5.39 ± 0.95	9.12	< 0.001
Problematic short video social media use (mean ± SD)		21.93 ± 6.84	18.35 ± 5.67	25.78 ± 5.82	21.79	< 0.001
Social anxiety (mean ± SD)		49.16 ± 10.20	46.93 ± 10.17	51.54 ± 9.69	7.81	< 0.001
Maladaptive cognitions toward social media use (mean ± SD)		32.34 ± 8.01	29.54 ± 7.74	35.33 ± 7.16	13.04	< 0.001

Before data collection, all participants received information about the study. The participants were asked to sign and return a paper-based informed consent document, and parental/guardian consent was requested by mail if one was a minor. Participants completed self-administered questionnaires on the *Wenjuanxing* platform anonymously in quiet classroom settings to help ensure confidentiality. They were informed of their right to withdraw at any time without explanation and of the protection of their privacy. The study protocol was reviewed and approved by the Research Ethics Board of Zhejiang Normal University (No. ZSRT2022014).

Participants reported the time they spent on social media by being asked “On average, how much time per day have you spent using social media during the past week?” The item was rated on a 6-point scale ranging from 1 (*less than 10 minutes*) to 6 (*more than 3 hours*). The participants reported using social media approximately “2-3 hours” per day (*M* = 5.08, *SD* = 1.14), with 89.7% of the students using social media more than 1 hour a day and only 0.7% using social media less than 10 minutes per day.

### 2.2 Measurements

#### 2.2.1 Problematic social media use

The Chinese version of the Bergen Social Media Addiction Scale (BSMAS) (17) was used to assess PSMU. The scale included six items examining the experience of social media usage over the past year (i.e., “How often during the past year have you …”). Each item reflected one of the six core addiction components (e.g., salience: “… spent a lot of time thinking about social media or planned use of social media?”) (14). Responses were rated on a 5-point scale ranging from 1 (*very rarely*) to 5 (*very often*), and the total score ranged from 6 to 30. Higher scores on the items suggested higher levels of specific components of PSMU. A confirmatory factor analysis (CFA) was performed using Mplus (version 8.3) to test the factor structure of this scale, supporting the one-factor solution (*χ* ^2^/*df* = 2.654, CFI = 0.994, TLI = 0.989, RMSEA = 0.038, SRMR = 0.015). Cronbach’s α for the present sample was 0.83.

#### 2.2.2 Problematic short video social media use

The problematic use of short video social media was measured using the adapted Chinese version of the Internet Gaming Disorder Scale-Short Form (IGDS-SF) (17). The IGDS-SF was modified by replacing the terms “gaming behavior” or “gaming activity” with “short video social media use” in the present study. Short video social media refers to websites or apps enabling online social interaction and the creation/sharing of short videos shorter than 5 minutes (e.g., TikTok) (36). This 9-item scale was developed based on the nine criteria for IGD depicted in the *Diagnostic and Statistical Manual of Mental Disorders*, 5th edition (DSM-5) (37). Responses were rated on a 5-point scale ranging from 1 (*never*) to 5 (*very often*), and the total score ranged from 9 to 45. Higher scores on the items indicated a greater likelihood of being at risk for developing specific components of problematic short video social media use. Given that scales developed based on the DSM-5 criteria for IGD have shown a robust single-factor structure (17, 38), we employed CFA to validate the structure of problematic short video social media use scale. The CFA revealed that a single-factor model provided a good fit to the data (*χ* ^2^/*df* = 4.361, CFI = 0.986, TLI = 0.974, RMSEA = 0.056, SRMR = 0.022). Cronbach’s α of the scale in this study was 0.90.

#### 2.2.3 Social anxiety

To assess social anxiety, the Chinese version of the Interaction Anxiousness Scale (39, 40) was utilized. The scale included 15 items (e.g., “I usually feel uncomfortable when I am in a group of people I don’t know”). Responses were rated on a 5-point scale ranging from 1 (*not at all*) to 5 (*extremely*), and the total score ranged from 15 to 75. In this study, scores were averaged across all items, with higher scores indicating greater levels of social anxiety. Cronbach’s α for the current sample was 0.88.

#### 2.2.4 Maladaptive cognitions toward social media use

The Chinese Online Maladaptive Cognitions Scale (41) was used to assess participants’ maladaptive cognitions toward social media use. The scale comprised 12 items (e.g., “Only friends on social media will tell you more sincere words”). Responses were rated on a 5-point scale ranging from 1 (*totally disagree*) to 5 (*totally agree*), and the total score ranged from 12 to 60. In this study, scores were averaged across all items, with higher scores signifying greater levels of maladaptive cognitions concerning social media use. Previous studies reported promising psychometric properties of this scale in adolescent (41) and young adult (42) social media users. Cronbach’s α for the present sample was 0.87.

### 2.3 Statistical analyses

#### 2.3.1 Data preparation

Network analyses were performed using *R*-version 4.3.2 (43). The network analysis approach and *R* code implemented in this study followed Heeren et al.’s study (33). The participants could not skip any items because of the option settings of the online survey. Thus, there were no missing data among the included participants. While initial normality assessments, based on the indices of skewness > |2| and kurtosis > |7| (44), did not indicate violations (see **Supplementary Table S1**), a nonparanormal transformation was applied to the six PSMU components via *huge* package (version 1.3.5) (45) in accordance with established methodological recommendations (33).

To evaluate the potential conceptual overlap among the variables within the network, a data-driven method (33) was employed to identify redundant nodes. This involved first confirming the positive definiteness of the correlation matrix for all variables within the network and then using the *goldbricker* function within *networktools* package (version 1.5.1) (46) to detect redundant variables. This analysis did not reveal any redundancies. As an additional measure, Unique Variable Analysis (UVA) was conducted using the *EGAnet* package (version 2.0.4) (47), identifying redundant node pairs based on a weighted topological overlap (wTO) exceeding 0.25 (48). This analysis identified one such pair, namely conflict and relapse (wTO = 0.29). However, the two components were not combined because they represent distinct aspects within the components model of addiction and correspond to different diagnostic criteria for behavioral addiction (IGD) within the DSM-5 (49).

To investigate the validity of the BSMAS, which uses single-item scores to assess each PSMU component, we calculated bivariate correlations between items of BSMAS and those of the problematic short video social media use scale using SPSS 22.0. Griffiths et al. (49) have proposed that components within the components model of addiction directly map onto the IGD criteria in the DSM-5. Specifically, each component within the components model of addiction matches a single IGD criterion in the DSM-5, except for the conflict component, which is captured in criteria 5, 6, 7, and 9 of the DSM-5 (49). Additionally, we examined the correlations between BSMAS items, time spent using social media, social anxiety, and maladaptive cognitions toward social media use. These analyses offered additional evidence for the validity of BSMAS.

#### 2.3.2 Estimation of the Gaussian graphical models

In alignment with recent recommendations (33, 50), the unregularized GGM network was estimated using the *ggmModSelect* algorithm within the *qgraph* package (version 1.9.8) (51). The decision to employ an unregularized model was based on the large sample size and the fact that the number of participants vastly exceeded the number of nodes (33, 50). In this procedure, the graphical LASSO (Least Absolute Shrinkage and Selection Operator) was utilized to create 100 regularized networks ranging from sparse to dense. Each of these 100 networks was then re-estimated *without* regularization through maximum likelihood estimation, retaining only non-zero edges from the regularized networks. The Bayesian information criterion (BIC) was computed iteratively for each newly estimated model, and the model exhibiting the lowest BIC was selected, signifying an optimal model where no further edge additions or removals could enhance model fit. In the GGM network, nodes represent components, and edges denote the partial correlations between them. The thickness of an edge visually conveys the strength of the correlation, which is described as the term of edge weight, with a thicker edge indicating a stronger connection. The Fruchterman and Reingold (52) algorithm was implemented for node placement, positioning more highly connected nodes in closer proximity. Additionally, a regularized GGM network was estimated to complement the unregularized model. This model was constructed using the graphical LASSO in combination with extended Bayesian information criterion (EBIC), calculated using the *EBICglasso* function within the *qgraph* package (version 1.9.8) (51). The graphical LASSO regularization allows for the shrinkage of small edge weights to zero, resulting in a sparser network. This process is controlled by adjusting a tuning hyperparameter (γ) between 0 (*less conservative*) and 1 (*more conservative*). In this analysis, γ was set to 0.5 to achieve a balance between sensitivity and specificity in the network structure (33). The results were almost identical to those resulting from *ggmModSelect* model (see **Supplementary Figures S1**, **S2**).

To assess the importance of nodes within the network, we calculated their strength centrality indices (29). Strength centrality is the sum of the absolute values of edge weights connected to a node (29). In our data, higher strength values represent potential greater centrality and importance within the network.

To evaluate the robustness of the network, we employed the *bootnet* package (version 1.5.6) (53). Edge weight stability was assessed using nonparametric bootstrapping with 1000 bootstrap samples to construct 95% confidence intervals (CIs). Narrower CIs indicate more precise edge estimations (53). Strength centrality stability was quantified using the correlation stability coefficient (CS-coefficient), calculated via case-dropping bootstrapping with 1000 bootstrap samples. This coefficient reflects the maximum proportion of cases that can be removed while preserving a strong correlation (> 0.70) between the original dataset’s centrality indices and those of subsets with a 95% probability (53). Acceptable CS-coefficients should not fall below 0.25, with values of 0.5 or higher being preferred (53). In addition, bootstrapped difference tests were conducted to examine variations in edge weights and node centrality indices via *bootnet* package (version 1.5.6).

To compare network differences between low- and high-BSMAS-score subgroups, we employed the *Network Comparison Test* package (version 2.2.2) (54), using 1000 iterations, and with seed set to “012”. Aligning with a recent meta-analysis (22), participants were categorized into two subgroups defined by the average of BSMAS sum-scores (*M* = 16.22, *SD* = 5.01). The low- and high-BSMAS-score subgroups comprised 588 and 548 students, respectively. We conducted three tests: a network structure invariance test, an edge invariance test, and a centrality invariance test (54). The network invariance test was evaluated by the maximum difference in the strength of the edges between the networks; it compares the overall pattern of connections between groups. The edge invariance test was evaluated by the differences in individual edge weights between the two networks. The centrality invariance test examined differences in the centrality strength of specific nodes across the networks. To visualize these networks with enhanced clarity, we utilized the *AverageLayout* function within the *qgraph* package (version 1.9.8).

#### 2.3.3 Directed acyclic graphs

To compute the DAGs, we employed a Bayesian hill-climbing algorithm within the *bnlearn* package (version 4.9.1) (55). This algorithm iteratively refines the network’s structure by adding, removing, or reversing edges to optimize the goodness-of-fit target score (i.e., Bayesian information criterion [BIC]). The algorithm initiates with a bootstrap function that estimates initial edge configurations. It then repeatedly adjusts edges to enhance BIC scores. To avoid getting trapped in local maxima, the algorithm incorporates 50 random restarts (33), each involving 100 edge perturbations (additions, removals, or reversals) (33). Throughout this iterative procedure of random restart/perturbation, the algorithm selects the model with the optimal BIC value and discloses the structure of the network.

Following recent guidelines (33, 56), we assessed the stability of the resultant DAGs as follows. We generated 10,000 network samples (with replacement), and with seed set to “012”. We averaged across the networks to construct a final, robust network structure through two steps. First, we analyzed the edge frequency across the bootstrapped networks. Specifically, we examined the proportion of samples in which each edge appeared. Employing the optimal cut-point method of Scutari and Nagarajan (57), we retained only edges exceeding a predetermined frequency threshold, ensuring both high sensitivity and specificity in the final network. Second, we evaluated the directionality of each retained edge. If an edge consistently pointed from node X to node Y in more than 50% of the bootstrapped networks, this direction was incorporated into the final network using an arrow (56).

Building upon previous research (32, 33), we employed two visualizations to depict the averaged network. In the first visualization, arrow thickness represents the BIC value of an arrow. The absolute BIC values indicate the importance of an arrow to the model fit. The thicker an arrow (and the higher its absolute BIC value), the more damaging its removal would be to the model fit. The second visualization uses arrow thickness to depict directional probabilities. Thicker arrows indicate a greater likelihood that the depicted direction is correct. Conversely, very thin arrows (closer to 50%) suggest that they have nearly equal likelihood to point in either direction, and they may represent simultaneous processes.

## 3 Results

### 3.1 Descriptive statistics and bivariate relationships

Descriptive statistics for the six PSMU components, including mean, standard deviation, range, skewness, and kurtosis, are presented in **Supplementary Table S1**. Pearson correlation analyses revealed that the six PSMU components were moderately and positively correlated with their corresponding counterparts in the scale of problematic short video social media use (*r* range = 0.316−0.531, *p*s < 0.001) (see **Supplementary Table S2**). Notably, these correlations were consistently stronger than those observed between PSMU components and most other components of the problematic short video social media use. Moreover, PSMU components had moderate, positive correlations with the duration of social media use (*r* range = 0.201−0.323, *p*s < 0.001), social anxiety (*r* range = 0.109−0.250, *p*s < 0.001), and maladaptive cognitions toward social media use (*r* range = 0.256−0.375, *p*s < 0.001). These results support the validity of the BSMAS as a measure of PSMU. In addition, compared to the low-BSMAS-score subgroup, the high-BSMAS-score subgroup exhibited significantly higher levels of duration of social media use (*t*_(1134)_ = 9.12, *p* < 0.001), problematic short video social media use (*t*_(1134)_ = 21.79, *p* < 0.001), social anxiety (*t*_(1134)_ = 7.81, *p* < 0.001), and maladaptive cognitions toward social media use (*t*_(1134)_ = 13.04, *p* < 0.001) (see **Table 1**).

### 3.2 Gaussian graphical models

**Figure 1:**
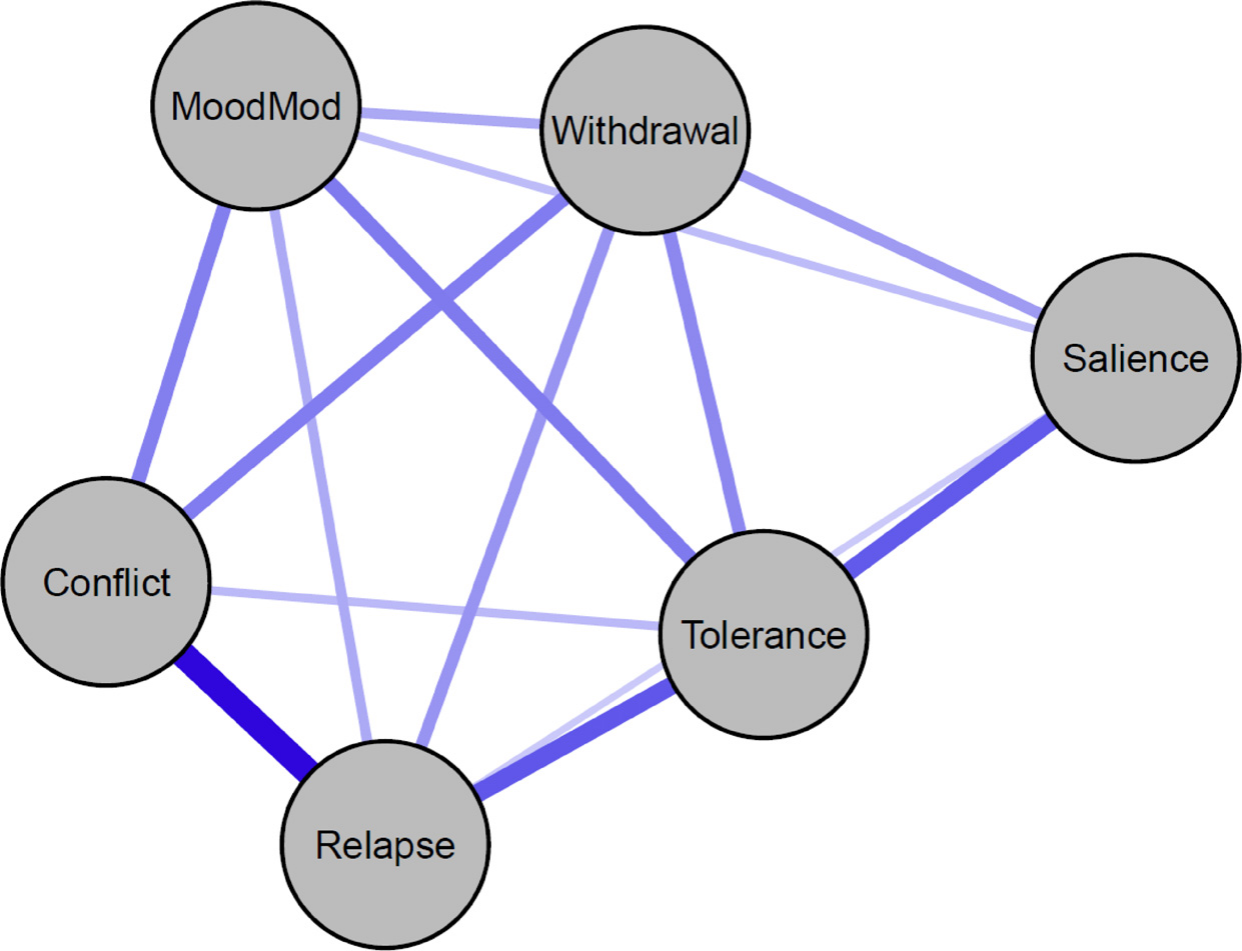
Network structure of problematic social media use components. MoodMod, Mood modification.

**Figure 1** depicts the unregularized GGM network. All edges displayed as blue and signified positive associations (negative associations, if any, would be represented in red). No certain minimum/maximum/cut values have been utilized when plotting the network. Edge weights ranged from 0.08 (relapse − salience) to 0.36 (relapse − conflict). The three strongest edges were observed between relapse and conflict (*r*_p_ = 0.36), relapse and tolerance (*r*_p_ = 0.24), and tolerance and salience (*r*_p_ = 0.24), respectively. The stability of the estimated edges was relatively good because the confidence intervals were narrow (see **Supplementary Figure S3**), and the strongest and weakest edges were significantly different from one another (see **Supplementary Figure S4**).

**Figure 2:**
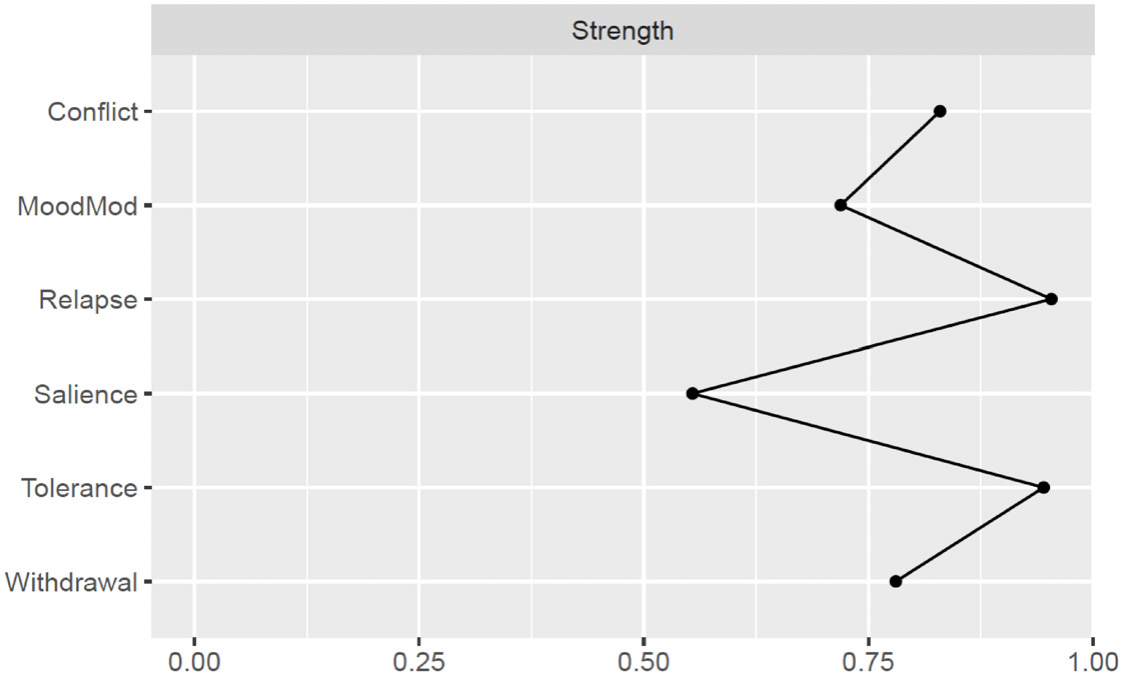
Strength centrality of each node in the problematic social media use network. MoodMod, Mood modification.

Strength estimates are illustrated in **Figure 2** and **Supplementary Table S3**. Relapse and tolerance components exhibited the highest strength values, while salience had the lowest. The stability of centrality estimates was found to be high (CS-coefficient = 0.75) (see **Supplementary Figure S5**).

Bootstrapped difference tests demonstrated that relapse and tolerance nodes were significantly more central than most other nodes, including withdrawal, mood modification, and salience (see **Supplementary Figure S6**). Moreover, the two-tailed Pearson correlation between the standard deviations for component severity and strength estimates of the six nodes was not significant (*r* = -0.42, *t*_(4)_ = -0.94, *p* = 0.40). This suggests that the observed centrality patterns are not driven by differences in variance among components.

**Figure 3:**
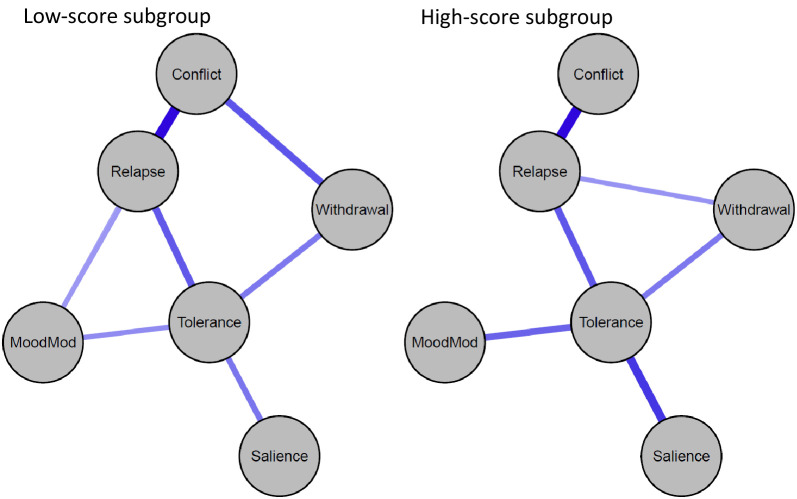
Problematic social media use networks for low-BSMAS-score subgroup (*n* = 588) and high-BSMAS-score subgroup (*n* = 548). Blue edges represent positive associations. MoodMod, Mood modification.

**Figure 4:**
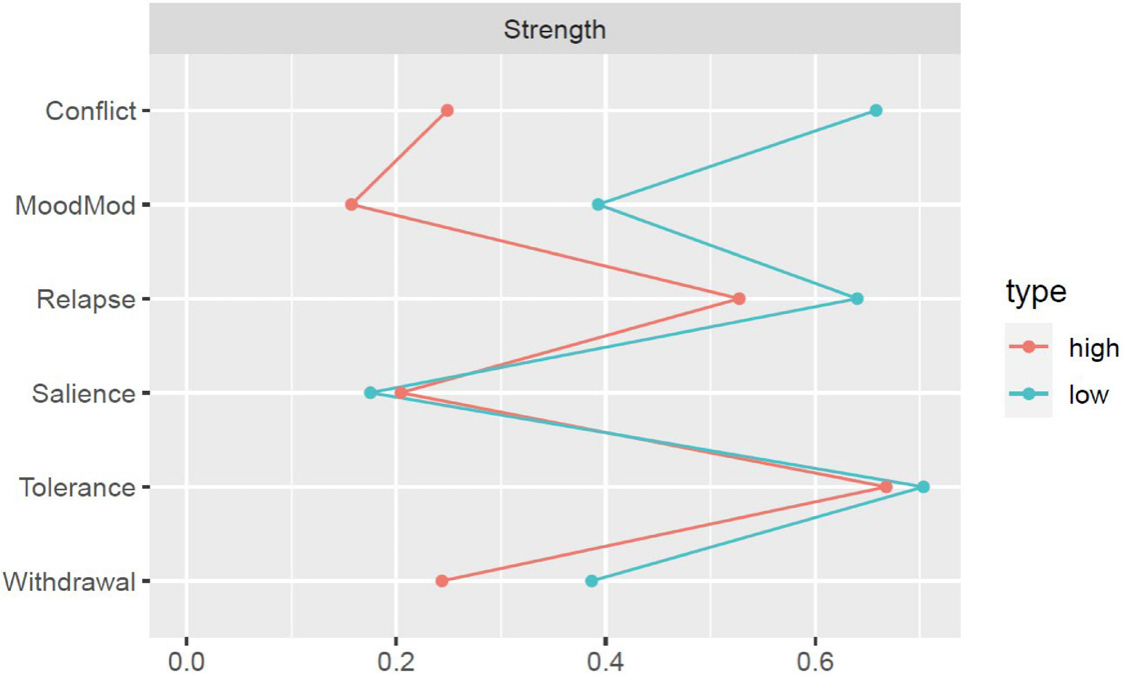
Strength centrality for problematic social media use components across subgroups with low and high BSMAS scores. MoodMod, Mood modification.

**Figures 3**, **4** illustrate the network structures and centrality indices for individuals with low and high BSMAS scores (see **Supplementary Figures S7**, **S8** for the stability of estimated edges and centrality indices of the two groups). We computed the density of the two networks by calculating the portion of estimated edges to the total number of possible edges. The network of high-BSMAS-score subgroup had a density of 0.40 (6/15 edges), with a mean weight of 0.07, and the low-BSMAS-score network had a density of 0.53 (8/15 edges), with a mean weight of 0.10. The network structure invariance test indicated no significant difference in the overall network structure between the groups (*M* = 0.22, *p* = 0.12), suggesting similar network configurations. However, the edge invariance test identified a significantly stronger link between conflict and withdrawal in the low-score subgroup compared to the high-score subgroup (*E* = 0.22, *p* = 0.01). The centrality invariance test revealed that low-score subgroup exhibited higher values of strength for conflict (*p* = 0.006) and mood modification (*p* = 0.047) compared to the high-score subgroup (see **Supplementary Table S3**). Moreover, the tolerance and relapse nodes held prominent positions in the network within the high-score subgroup.

### 3.3 Directed acyclic graphs

**Figure 5:**
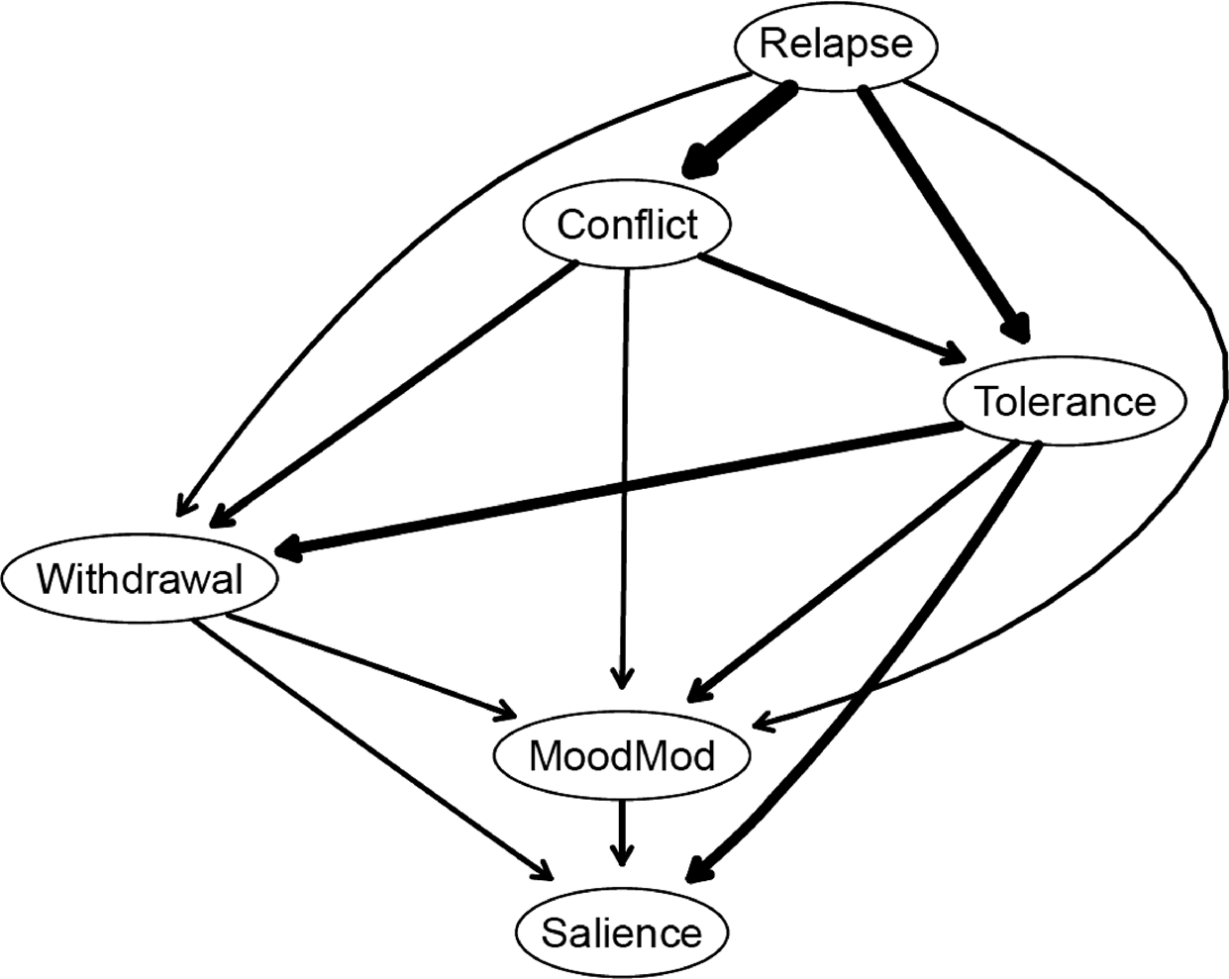
Bayesian network displayed as a directed acyclic graph (DAG). Components are presented as nodes with arrow thickness representing the importance of that arrow for the model fit. Greater thickness implies larger contribution to the model fit. MoodMod, Mood modification.

**Figure 6:**
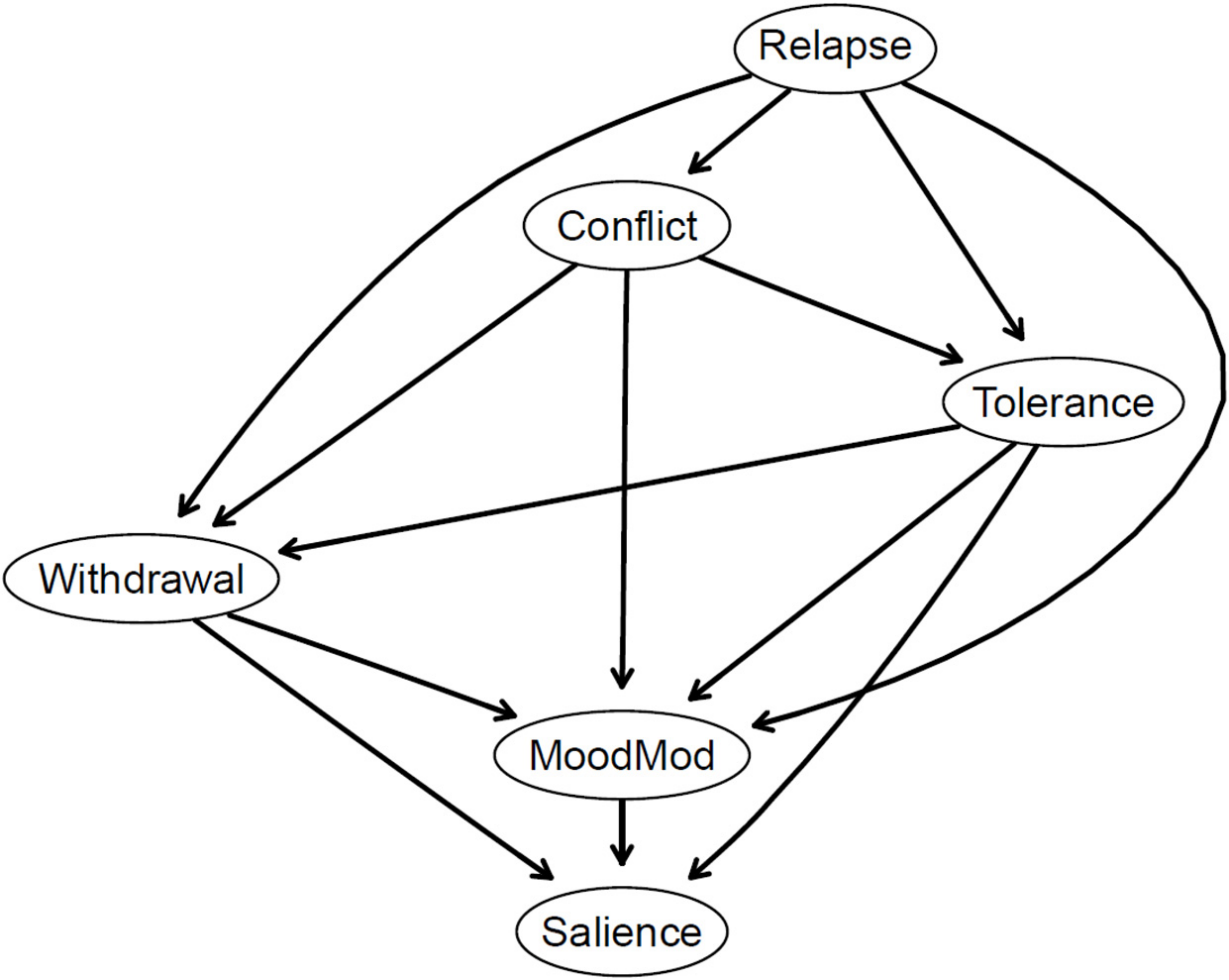
Bayesian network displayed as a directed acyclic graph (DAG). Components are presented as nodes with arrow thickness signifies the probability in the direction of prediction depicted. Greater thickness denotes higher proportions of the bootstrapped networks in which the arrow points in that direction. MoodMod, Mood modification.

**Figures 5**, **6** show the DAGs arising from 10,000 bootstrapped networks. In both DAGs, only edges with a magnitude exceeding the threshold determined by the Scutari and Nagarajan (57) method are included in the graphs.

In **Figure 5**, the thickness of arrows represents the change in BIC when the arrow is removed from the network. The most influential arrow in the network structure linked relapse to conflict. Additionally, arrows connecting relapse to tolerance as well as tolerance to salience also played crucial roles in shaping the network structure. The detailed information on the change in BIC values for each arrow is depicted in **Supplementary Table S4**.

In **Figure 6**, the thickness of arrows illustrates directional probabilities, representing the proportion of the 10,000 bootstrapped networks wherein the arrow points in a specific direction. All arrows exhibited similarly low directional probabilities (see **Supplementary Table S4**). The thickest arrow, connecting tolerance to salience, indicates that this directional connection occurred in 53.5% of the bootstrapped networks. The second and third thickest arrows pointed from withdrawal to salience (52.8%) and from tolerance to mood modification (51.5%), respectively, suggesting the potential for simultaneous processes between these components.

Structurally, the relapse component was positioned at the top of the DAGs, and interconnected with other components of PSMU. This implies that the occurrence of tolerance, conflict, withdrawal, mood modification, and salience is likely to co-occur with the presence of relapse. Additionally, several nodes may serve as key intermediate steps within the cascading model. These include tolerance (with two incoming and three outgoing connections), conflict (with one incoming and three outgoing connections), withdrawal (with three incoming and two outgoing connections), and mood modification (with four incoming and three outgoing connections).

Of note, the DAGs were overall sparser (13 edges) than the GGMs (14 edges) (see **Figure 1**; **Supplementary Figure S1**). The GGMs included an additional edge (relapse − salience) not present in the DAGs, and this edge had the smallest edge weight (*r*_p_ = 0.08). Moreover, arrows with larger absolute BIC values (and with greater thickness) in the DAG (see **Figure 5**) tended to match with edges having larger edge weights in the GGMs. In other words, the edges included in the DAGs appeared to be strong edges in the GGMs.

## 4 Discussion

To examine the network structure of PSMU, this study computed regularized and nonregularized GGMs and Bayesian DAGs through state-of-the-art analytic guidelines (51, 56) in a large sample of Chinese college students reporting a history of social media use.

### 4.1 Central components and component relationships of PSMU

In our initial analysis, we examined the validity of the Chinese version of the BSMAS. The results revealed that all items on the BSMAS exhibited moderately positive correlations with their corresponding dimensions on the problematic short video social media use scale. These correlations were stronger than those between BSMAS items and most other components of problematic short video social media use. Additionally, all BSMAS items showed moderate, positive correlations with time spent using social media, social anxiety, and maladaptive cognitions concerning social media use. These findings align with our hypothesis (H1) and previous studies (17, 18), suggesting that the BSMAS is a valid instrument to assess the magnitude of PSMU components among Chinese college students.

Across our analyses of the whole sample, findings highlighted the importance of the items representing relapse and tolerance, while revealing the periphery role of salience within the PSMU network. In the GGMs, relapse and tolerance nodes yielded the highest strength, whereas the salience node exhibited the lowest value. Moreover, the DAGs positioned relapse as the parent node at the top of the cascading network, indicating its frequent co-occurrence with other PSMU components (32). Notably, these two nodes were strongly interconnected in both GGMs and DAGs and were linked to the most vital edges in the networks (e.g., edges connecting relapse to conflict; edges linking tolerance to salience).

These findings align with our hypothesis (H2) and previous network studies of PSMU (24, 26). For instance, Svicher et al. (24) identified the item representing relapse as a highly central node in the PSMU network among college students. They also found that the items denoting preference for online social interaction were highly influential. This discrepancy between studies might be due to differences in PSMU measurement and sample characteristics. Additionally, Li et al. (26) found the item representing tolerance to be central and salience to be periphery within the PSMU network. However, other network studies have classified tolerance (and salience) as peripheral rather than central PSMU components (5, 27), indicating a need for further investigation. Our findings support the iRISA model of addiction (34). This model posits that the decreased inhibitory control and heightened incentive salience attributed to drugs or drug-related cues contribute to the development of addiction symptoms like craving, withdrawal, and bingeing. Combined with our results, relapse may emerge as a core feature in the context of PSMU, potentially influencing the activation of the rest of the PSMU network when triggered (24).

The DAGs also support our hypothesis (H2) and provide complementary insight into the associations among PSMU components. Specifically, the item representing relapse emerged as the parent node, predicting tolerance, conflict, withdrawal, and mood modification, which subsequently predicted salience across the entire sample. This pattern suggests that the presence of salience, mood modification, withdrawal, conflict, and tolerance is likely to accompany the existence of the relapse component. One plausible explanation is that when students experience decreased reflective or controlled functions, they might fail to focus on study/work tasks or efficiently interact with others, spend an ever-increasing amount of time engaging in social media usage to reach desired effects, feel restless or irritable when offline, and turn to further social media use to increase pleasure and alleviate discomfort (58). Ultimately, they might prioritize social media use over other activities. However, the directional probabilities of the DAG ranged from 0.501 to 0.535 (see **Supplementary Table S4**), suggesting that the arrows had nearly equal likelihood of pointing in either direction for this specific dataset. This bidirectional prediction between the components decreases our confidence in determining the parent and offspring components. Therefore, the present results should be interpreted with caution. In addition, the DAGs were sparser than the GGMs. The edge (relapse − salience) with the smallest edge weight in the GGMs was absent in the DAGs. Moreover, arrows with larger absolute BIC values in the DAG tended to correspond with edges having larger edge weights in the GGMs. These findings align with previous network studies (32, 33, 59), suggesting a broad agreement between DAGs and GGMs on the most important edges in the network (59).

The current study found that participants with high BSMAS scores exhibited significantly higher levels of social media and short video social media use behaviors, social anxiety, and maladaptive cognitions concerning social media use compared to those with low BSMAS scores. These findings concur with previous research (22), validating the use of BSMAS mean scores to classify participants. Finally, despite the overall network structure remaining similar between the high- and low-BSMAS-score subgroups, the high-score subgroup exhibited a sparser network and lower edge strength compared to the low-score subgroup. This pattern deviates from network theory, which often predicts stronger network connectivity in groups experiencing more severe stages (25). However, this phenomenon has been observed in other network studies of mental disorders (60–62), highlighting the need for further exploration. Notably, the node strength of both conflict and mood modification was higher in the low-score subgroup compared to the high-score subgroup, supporting H3. In the high-score subgroup, items representing tolerance and relapse held prominent positions in the network, consistent with findings from IGD studies (28). One potential explanation for this shift may partly lie in Chinese Confucian culture, which emphasizes filial piety and avoiding bringing shame to the family (63). College students in this cultural setting often face significant pressures related to academic competition, interpersonal relationships, and family expectations (3). To cope with these pressures, they may use social media increasingly and feel that social media use affects their studies or work. Over time, with continued use, individuals may develop tolerance and relapse components due to diminished risk perception and heightened sensation seeking in social media use (28, 64).

### 4.2 Practical implications

Network theory posits that central nodes play a pivotal role in the occurrence or maintenance of mental disorders (65). From this perspective, activating highly central nodes can trigger other nodes via direct or indirect paths, promoting a cascade of node activation (65). Conversely, “turning off” the key nodes through targeted interventions could potentially suppress activation throughout the network, thereby disrupting the vicious cycle of components (65). Previous intervention research has adopted comprehensive strategies, such as cognitive behavioral therapy (CBT), to address PSMU as a whole, showing alleviation of PSMU components among adult social media users (66). Building upon network theory and our findings, interventions focusing on components with high centrality, such as mood modification and conflict, might be more effective in preventing PSMU development among college students with low BSMAS scores. Practitioners can implement strategies that encourage college students to engage in alternative offline interests (e.g., physical activity participation) and social activities (e.g., spending more time with friends and family) (67), as well as conflict management techniques (e.g., exercises to balance social media use and academics) (68).

For college students with high BSMAS scores, while the validity of tolerance as a diagnostic criterion for behavioral addictions remains controversial and warrants further investigation (69), interventions targeting relapse may present a promising avenue for decreasing the occurrence of PSMU. College mental health counselors often employ programs such as group counseling and psychoeducation to intervene in college students’ PSMU. However, these programs rarely target specific components of PSMU (70). Based on our results, mental health counselors could use strategies to strengthen students’ self-control processes. For example, they could train students to manage the feelings and thoughts that trigger excessive social media engagement, implement temptation-overcoming strategies, and perform daily practices like setting and adhering to time limits (71).

### 4.3 Limitations and future directions

The present study had some limitations. First, the estimation of both the GGMs and DAGs rested on cross-sectional data, which precluded any strong inference about the potential causal associations between PSMU components. Indeed, the present DAGs can only be interpreted as a product of each component’s conditional probability distribution given its parent nodes within the estimated model (33). Therefore, they cannot reveal temporal precedence between components. Recently, many studies have used longitudinal designs to examine the causal relationships between PIU (29, 31) or problematic smartphone use (PSU) (30) components. For example, Huang et al. (30) identified the indicator “continued excessive smartphone use” as a key driver within the PSU network over time. Second, while arrows with low directional probabilities may suggest potential feedback cycles between components, the DAGs approach cannot reveal cyclical dynamics within the network. This clinical dynamic may be an important feature of mental disorder network structures (25). Future research can leverage alternative methods (e.g., experience sampling method and time series analysis) to unravel the causal relationships and reinforcement loops between PSMU components within a temporal network (65).

Third, given the absence of established cut-off values for PSMU in Chinese college student social media users, this study compared networks between subgroups defined by BSMAS sum-scores. This approach might potentially introduce bias into the network structures. However, according to Haslbeck et al.’s (72) study, the estimator of this study (i.e., the sample networks of participants identified and not identified as PSMU) tended to correspond with the target of inference (i.e., the networks within college students identified and not identified as PSMU). As such, the bias introduced here might be small. Nevertheless, future studies should establish diagnostic cut-off scores for the BSMAS in this population to further validate and extend our findings.

Finally, the BSMAS items are general in nature due to its reliance on the components model of addiction. Therefore, this measure may not fully capture facets unique to PSMU, such as preference for online social interaction (24). Additionally, healthy young college students were recruited, so the findings cannot be generalized to adolescents, older adults, and clinical populations. Future studies should analyze the component structure of PSMU using more multifaceted instruments (12, 24) in conjunction with the BSMAS among more diverse samples to provide greater insight into the mechanisms underlying PSMU.

## 5 Conclusions

The present findings highlight relapse (and tolerance) as key components in the network system of PSMU across the entire sample as well as in the high-BSMAS-score subsample. Compared to the high-BSMAS-score subgroup, conflict and mood modification nodes appear to play more influential roles in the PSMU network within the low-BSMAS-score subgroup. Future intervention studies can concentrate on specific PSMU components based on severity levels to maximize the effectiveness of interventions. These efforts could improve our understanding of the network structure of PSMU and offer a complementary way in the quest to relieve PSMU among college students.

## Data Availability

The raw data supporting the conclusions of this article will be made available by the authors, without undue reservation.
