# Supplementary material for: Network modeling of problematic social media use components in college student social media users
Source: Front Psychiatry. 2025 Jan 13;15:1386845. doi: 10.3389/fpsyt.2024.1386845 (PMC11770832; doi:10.3389/fpsyt.2024.1386845)

**Network modeling of problematic social media use components in college student social media users**

Supplementary materials

**Table S1.** Mean, Standard Deviation (SD), Minimum (Min), Maximum (Max), Skewness, and Kurtosis of each component of problematic social media use (PSMU).

| Components | Mean | SD | Min | Max | Skewness | Kurtosis |
| --- | --- | --- | --- | --- | --- | --- |
| Salience | 2.93 | 1.11 | 1 | 5 | -0.02 | -0.74 |
| Tolerance | 2.94 | 1.14 | 1 | 5 | -0.06 | -0.84 |
| Mood modification | 2.64 | 1.18 | 1 | 5 | 0.22 | -0.84 |
| Relapse | 2.54 | 1.14 | 1 | 5 | 0.27 | -0.76 |
| Withdrawal | 2.75 | 1.17 | 1 | 5 | 0.12 | -0.83 |
| Conflict | 2.43 | 1.08 | 1 | 5 | 0.42 | -0.49 |

**Table S2.** Bivariate correlations among study variables.

| Item | 1 | 2 | 3 | 4 | 5 | 6 | 7 | 8 | 9 | 10 | 11 | 12 | 13 | 14 | 15 | 16 | 17 |
| --- | --- | --- | --- | --- | --- | --- | --- | --- | --- | --- | --- | --- | --- | --- | --- | --- | --- |
| 1. BSMAS‒Salience |  |  |  |  |  |  |  |  |  |  |  |  |  |  |  |  |  |
| 2. BSMAS‒Tolerance | 0.446^***^ |  |  |  |  |  |  |  |  |  |  |  |  |  |  |  |  |
| 3. BSMAS‒Mood modification | 0.344^***^ | 0.480^***^ |  |  |  |  |  |  |  |  |  |  |  |  |  |  |  |
| 4. BSMAS‒Relapse | 0.366^***^ | 0.545^***^ | 0.461^***^ |  |  |  |  |  |  |  |  |  |  |  |  |  |  |
| 5. BSMAS‒Withdrawal | 0.377^***^ | 0.487^***^ | 0.425^***^ | 0.489^***^ |  |  |  |  |  |  |  |  |  |  |  |  |  |
| 6. BSMAS‒Conflict | 0.341^***^ | 0.473^***^ | 0.463^***^ | 0.589^***^ | 0.476^***^ |  |  |  |  |  |  |  |  |  |  |  |  |
| 7. PSVSMUS‒Preoccupation/ Salience | **0.316^***^** | 0.426^***^ | 0.352^***^ | 0.375^***^ | 0.399^***^ | 0.359^***^ |  |  |  |  |  |  |  |  |  |  |  |
| 8. PSVSMUS‒Withdrawal | 0.275^***^ | 0.400^***^ | 0.379^***^ | 0.405^***^ | **0.475^***^** | 0.374^***^ | 0.580^***^ |  |  |  |  |  |  |  |  |  |  |
| 9. PSVSMUS‒Tolerance | 0.316^***^ | **0.400^***^** | 0.416^***^ | 0.395^***^ | 0.398^***^ | 0.346^***^ | 0.591^***^ | 0.618^***^ |  |  |  |  |  |  |  |  |  |
| 10. PSVSMUS‒Loss of control/ Relapse | 0.271^***^ | 0.398^***^ | 0.384^***^ | **0.531^***^** | 0.404^***^ | 0.447^***^ | 0.533^***^ | 0.597^***^ | 0.560^***^ |  |  |  |  |  |  |  |  |
| 11. PSVSMUS‒Giving up other activities | 0.254^***^ | 0.350^***^ | 0.355^***^ | 0.430^***^ | 0.328^***^ | **0.447^***^** | 0.450^***^ | 0.562^***^ | 0.496^***^ | 0.522^***^ |  |  |  |  |  |  |  |
| 12. PSVSMUS‒Continuing despite harms | 0.227^***^ | 0.345^***^ | 0.337^***^ | 0.372^***^ | 0.365^***^ | **0.378^***^** | 0.453^***^ | 0.540^***^ | 0.535^***^ | 0.494^***^ | 0.611^***^ |  |  |  |  |  |  |
| 13. PSVSMUS‒Deception | 0.250^***^ | 0.293^***^ | 0.299^***^ | 0.398^***^ | 0.321^***^ | **0.402^***^** | 0.377^***^ | 0.479^***^ | 0.459^***^ | 0.513^***^ | 0.524^***^ | 0.516^***^ |  |  |  |  |  |
| 14. PSVSMUS‒Escapism/Mood modification | 0.209^***^ | 0.372^***^ | **0.460^***^** | 0.380^***^ | 0.376^***^ | 0.357^***^ | 0.510^***^ | 0.476^***^ | 0.476^***^ | 0.519^***^ | 0.423^***^ | 0.463^***^ | 0.430^***^ |  |  |  |  |
| 15. PSVSMUS‒Impaired function | 0.219^***^ | 0.267^***^ | 0.295^***^ | 0.335^***^ | 0.288^***^ | **0.389^***^** | 0.293^***^ | 0.484^***^ | 0.435^***^ | 0.429^***^ | 0.589^***^ | 0.540^***^ | 0.606^***^ | 0.347^***^ |  |  |  |
| 16. Social anxiety | 0.109^***^ | 0.227^***^ | 0.216^***^ | 0.225^***^ | 0.246^***^ | 0.250^***^ | 0.175^***^ | 0.120^***^ | 0.133^***^ | 0.193^***^ | 0.148^***^ | 0.124^***^ | 0.152^***^ | 0.245^***^ | 0.058^*^ |  |  |
| 17. Maladaptive cognitions toward social media | 0.256^***^ | 0.339^***^ | 0.375^***^ | 0.314^***^ | 0.368^***^ | 0.322^***^ | 0.366^***^ | 0.417^***^ | 0.438^***^ | 0.334^***^ | 0.395^***^ | 0.422^***^ | 0.323^***^ | 0.397^***^ | 0.364^***^ | 0.225^***^ |  |
| 18. Duration of social media use | 0.232^***^ | 0.323^***^ | 0.201^***^ | 0.248^***^ | 0.260^***^ | 0.202^***^ | 0.269^***^ | 0.201^***^ | 0.219^***^ | 0.225^***^ | 0.140^***^ | 0.162^***^ | 0.122^***^ | 0.218^***^ | 0.079^**^ | 0.101^***^ | 0.131^***^ |

*Note*. *BSMAS* Bergen Social Media Addiction Scale, *PSVSMUS* Problematic Short Video Social Media Use Scale. The bold numbers represent the correlation coefficients between the components of BSMAS and the corresponding items of PSVSMUS. ^*^*p* < 0.05; ^**^*p* < 0.01; ^***^*p* < 0.001.

**Table S3.** Strength centrality values of the PSMU networks across the entire sample, low- and high-BSMAS-score subgroups.

| Components | Entire sample | Low-BSMAS-score subgroup | High-BSMAS-score subgroup |
| --- | --- | --- | --- |
| Salience | 0.55 | 0.18 | 0.20 |
| Tolerance | 0.95 | 0.70 | 0.67 |
| Mood modification | 0.72 | 0.39 | 0.16 |
| Relapse | 0.95 | 0.64 | 0.53 |
| Withdrawal | 0.78 | 0.39 | 0.24 |
| Conflict | 0.83 | 0.66 | 0.25 |

**Table S4.** Directional probabilities and BIC values of the arrows in directed acyclic graphs (DAGs) in the entire sample.

| Arrow in the DAG | | Value determining arrow thickness | |
| --- | --- | --- | --- |
| From | To | BIC | Directional Probability |
| Tolerance | Salience | -42.273 | 0.535 |
| Tolerance | Mood modification | -25.479 | 0.515 |
| Tolerance | Withdrawal | -34.288 | 0.506 |
| Mood modification | Salience | -4.282 | 0.512 |
| Relapse | Tolerance | -82.925 | 0.501 |
| Relapse | Mood modification | -6.230 | 0.512 |
| Relapse | Withdrawal | -17.025 | 0.506 |
| Relapse | Conflict | -243.464 | 0.505 |
| Withdrawal | Salience | -13.378 | 0.528 |
| Withdrawal | Mood modification | -7.845 | 0.507 |
| Conflict | Tolerance | -24.357 | 0.501 |
| Conflict | Mood modification | -16.151 | 0.510 |
| Conflict | Withdrawal | -25.130 | 0.502 |

*Note.* BIC quantifies the change in Bayesian Information Criterion upon removing an arrow from the network. Negative BIC values indicate decreases in the network score with the arrow’s removal. Stated differently, negative values signify enhanced model fit when the arrow is included. The directional probability values reflect the proportion of the bootstrapped networks where the arrow was present in a specific direction.

**Fig. S1.** The PSMU network generated by the graphical LASSO algorithm in the entire sample.


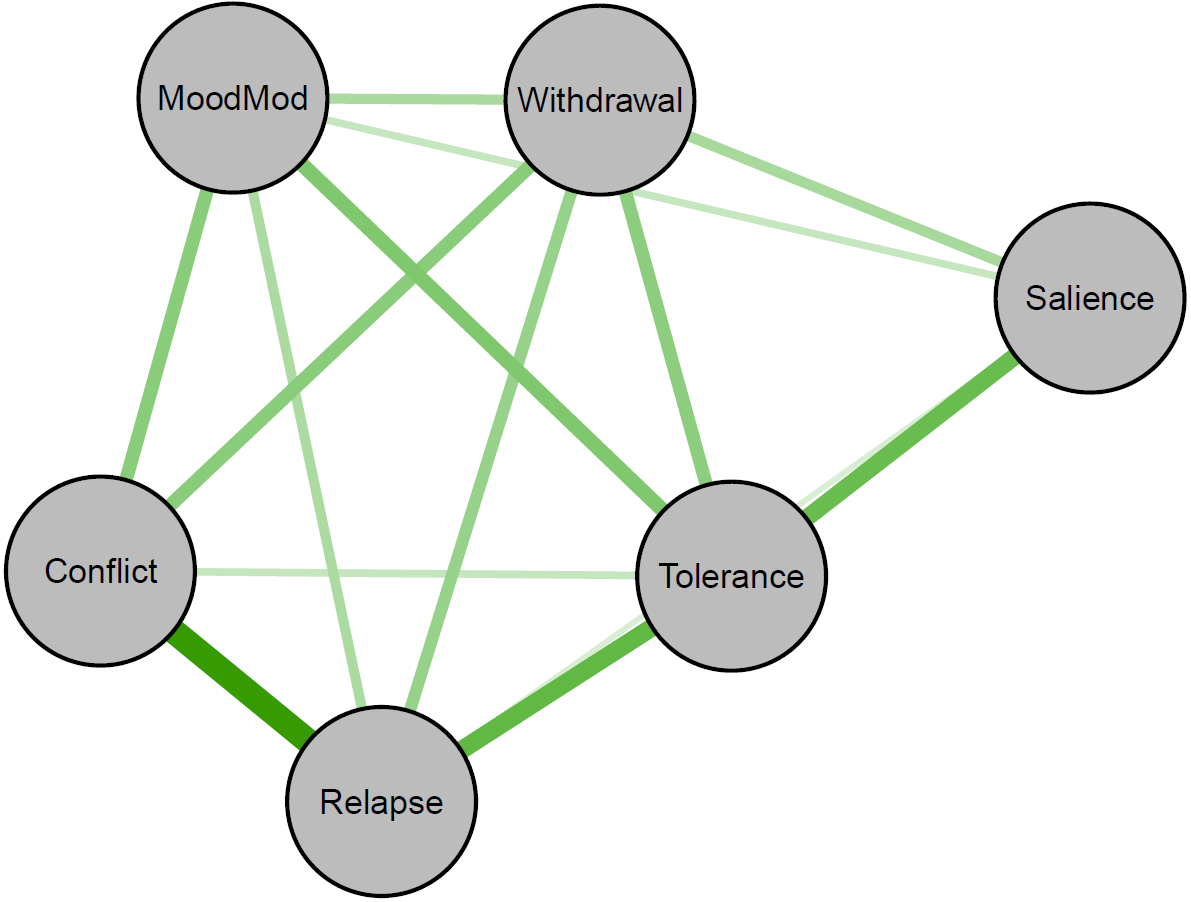


*Note.* All edges display as green and represent positive associations. Edge thickness signifies the association’s magnitude, with the thickest edge having a value of 0.35. MoodMod = Mood modification.

**Fig. S2.** Strength estimates of the PSMU network generated by graphical LASSO algorithm in the entire sample.


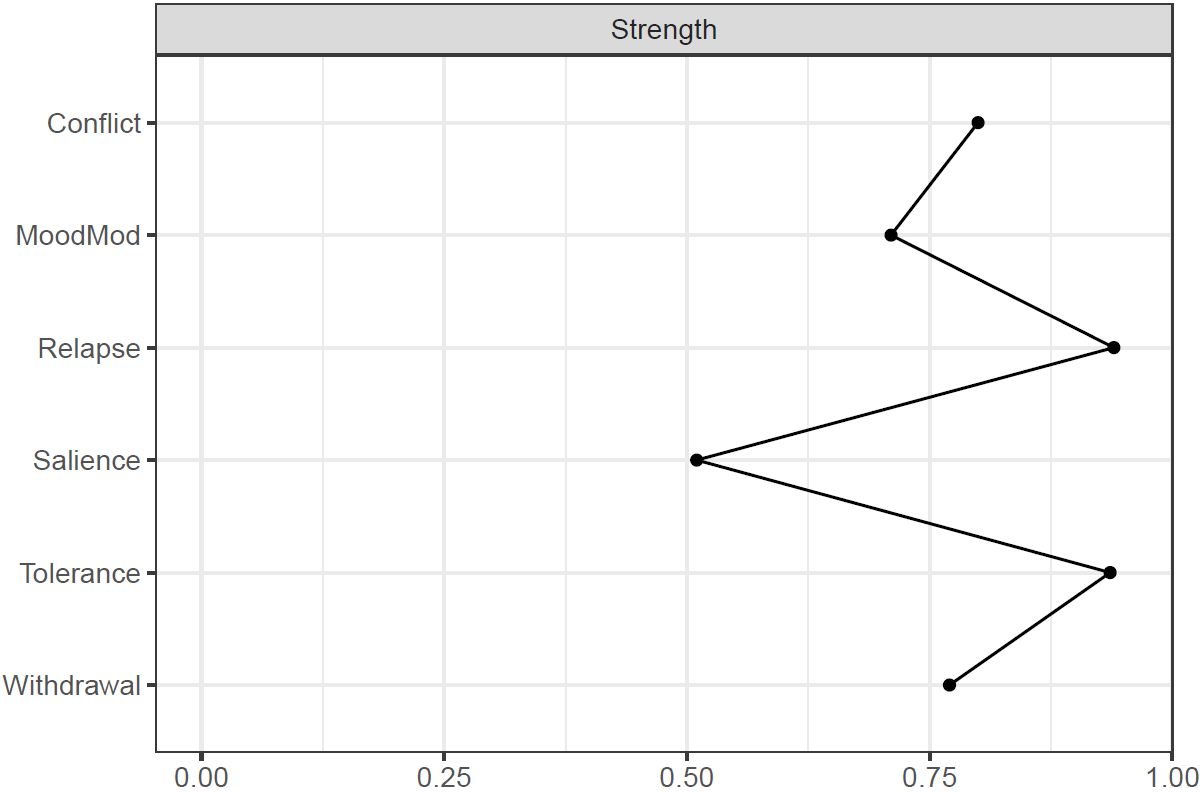


*Note.* MoodMod = Mood modification.

**Fig. S3.** Bootstrapped confidence intervals of estimated edge weights for the PSMU network generated by the ggmModSelect algorithm in the entire sample.


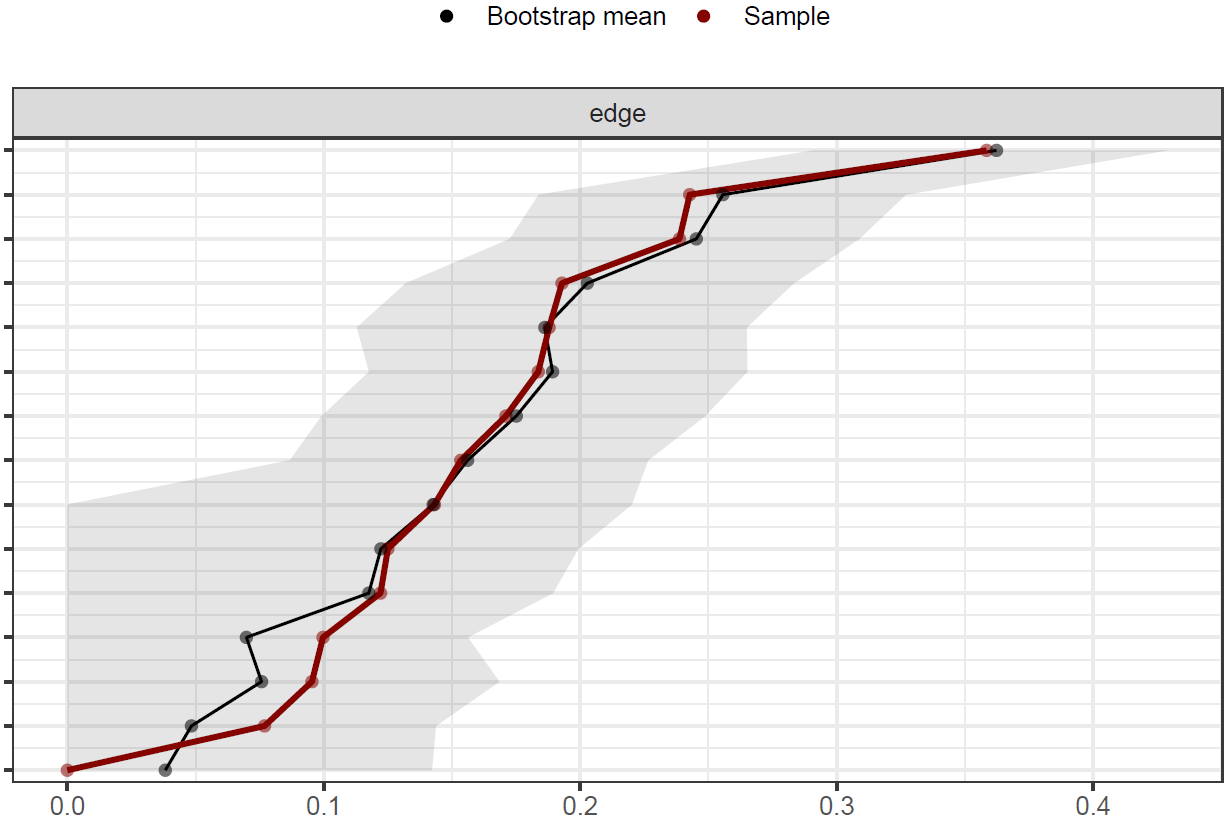


*Note.* The red line represents the values observed in the original sample, while the black line represents the mean bootstrapped values. The gray area denotes the 95% confidence intervals.

**Fig. S4.** Bootstrapped difference tests for edge-weights in the PSMU network generated by the ggmModSelect algorithm in the entire sample.


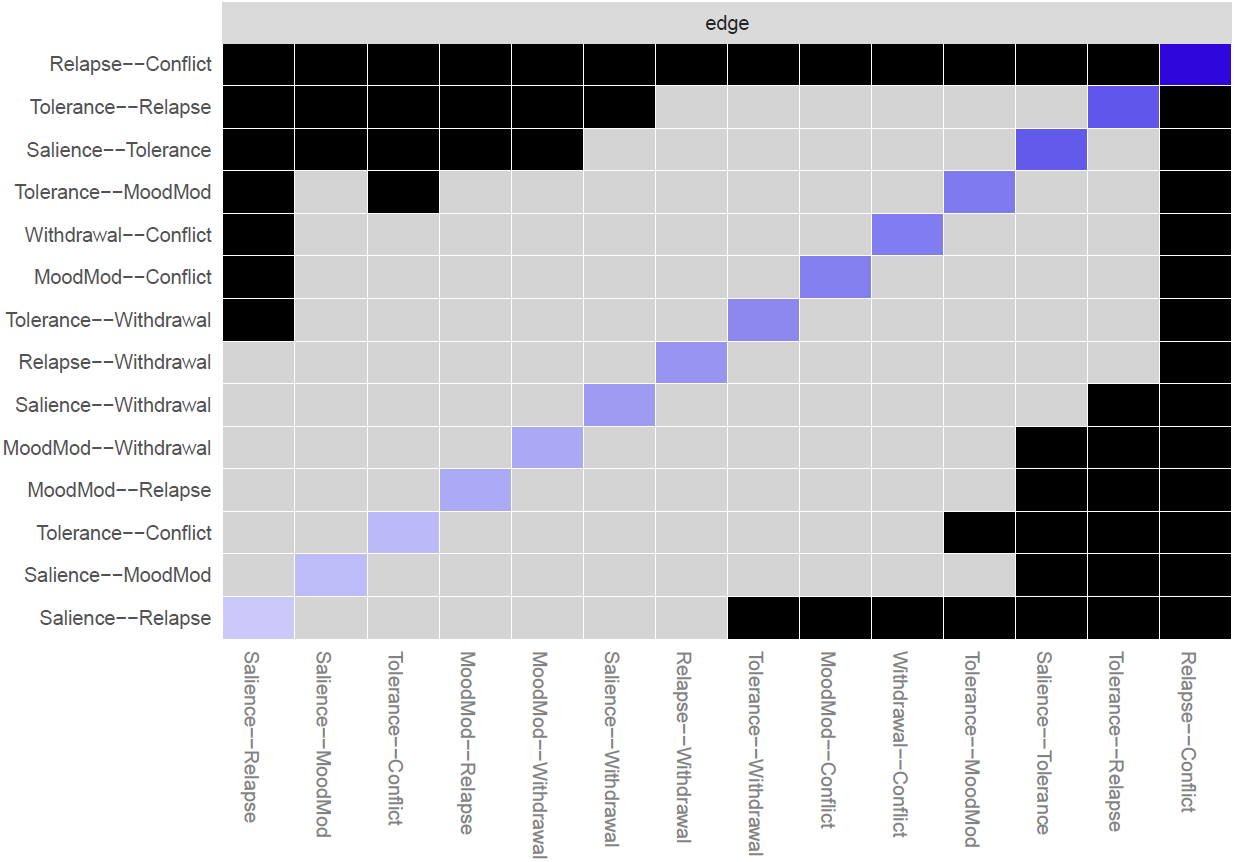


*Note.* Black squares signify statistically significant differences, while gray squares denote insignificant ones. The diagonal squares shaded in blue represent partial correlations between each node pair, with darker shades representing stronger associations. MoodMod = Mood modification.

**Fig. S5.** Stability of strength centrality for the PSMU network in the entire sample.


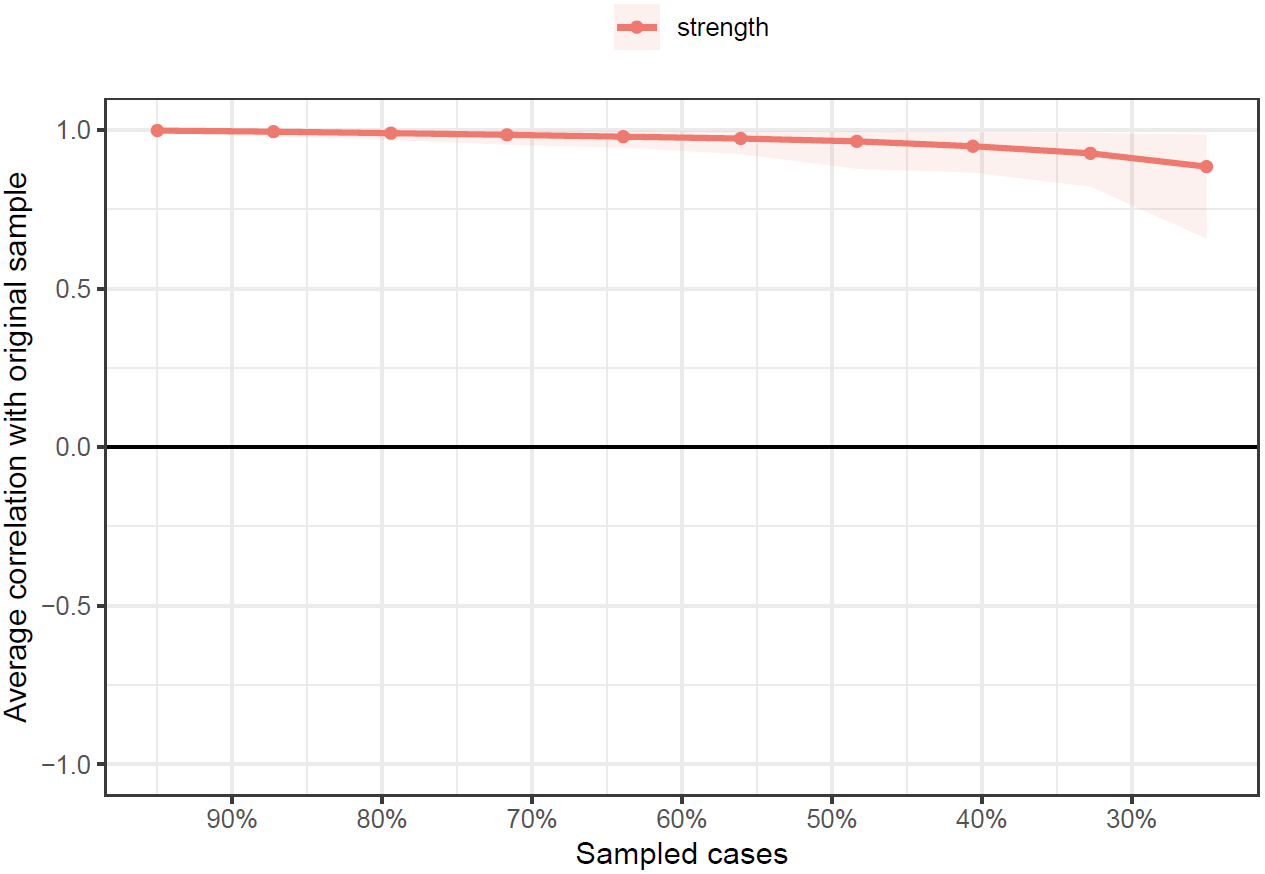


**Fig. S6.** Bootstrapped centrality difference tests for the PSMU network in the entire sample.


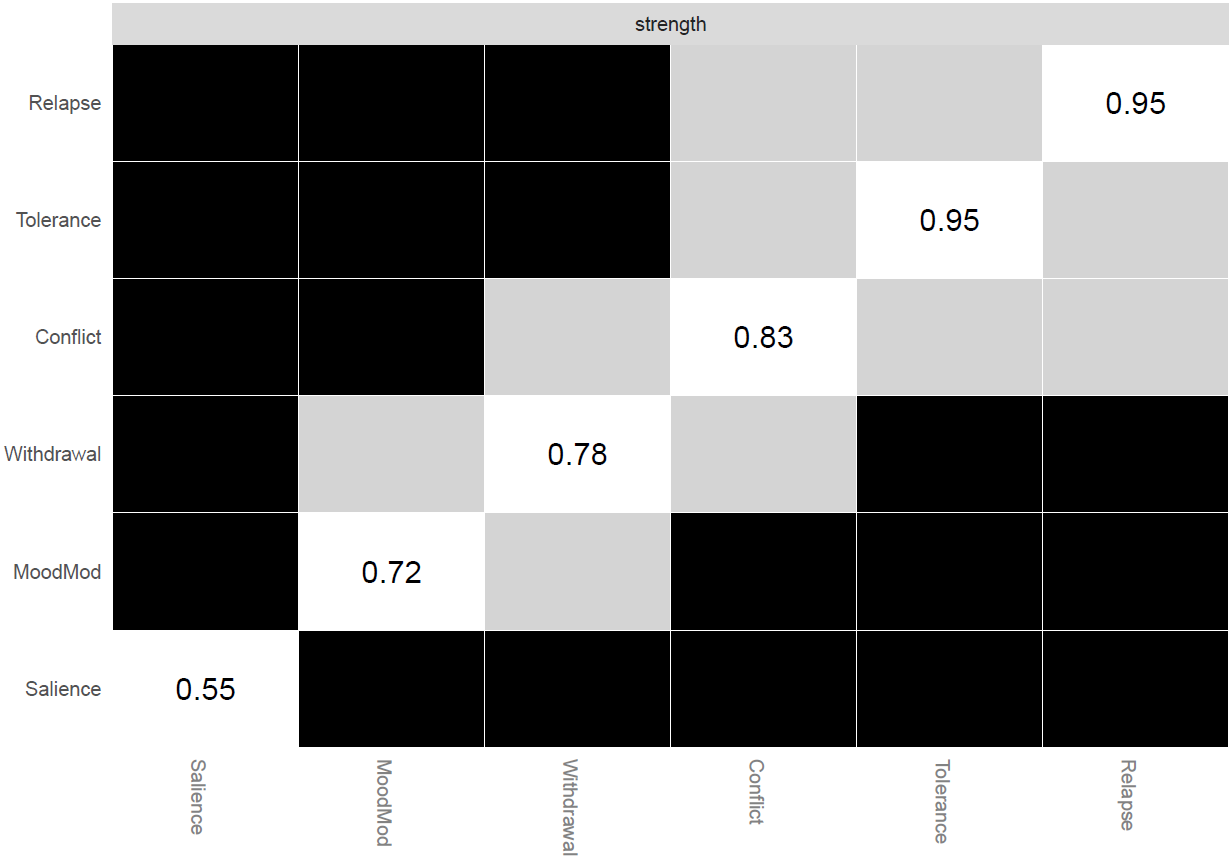


*Note.* Black squares represent significant differences, while gray squares denote insignificant ones. MoodMod = Mood modification.

**Fig. S7.** Bootstrapped confidence intervals of estimated edge weights for the PSMU network, generated by the ggmModSelect algorithm, in participants with low (top) and high (bottom) BSMAS scores.


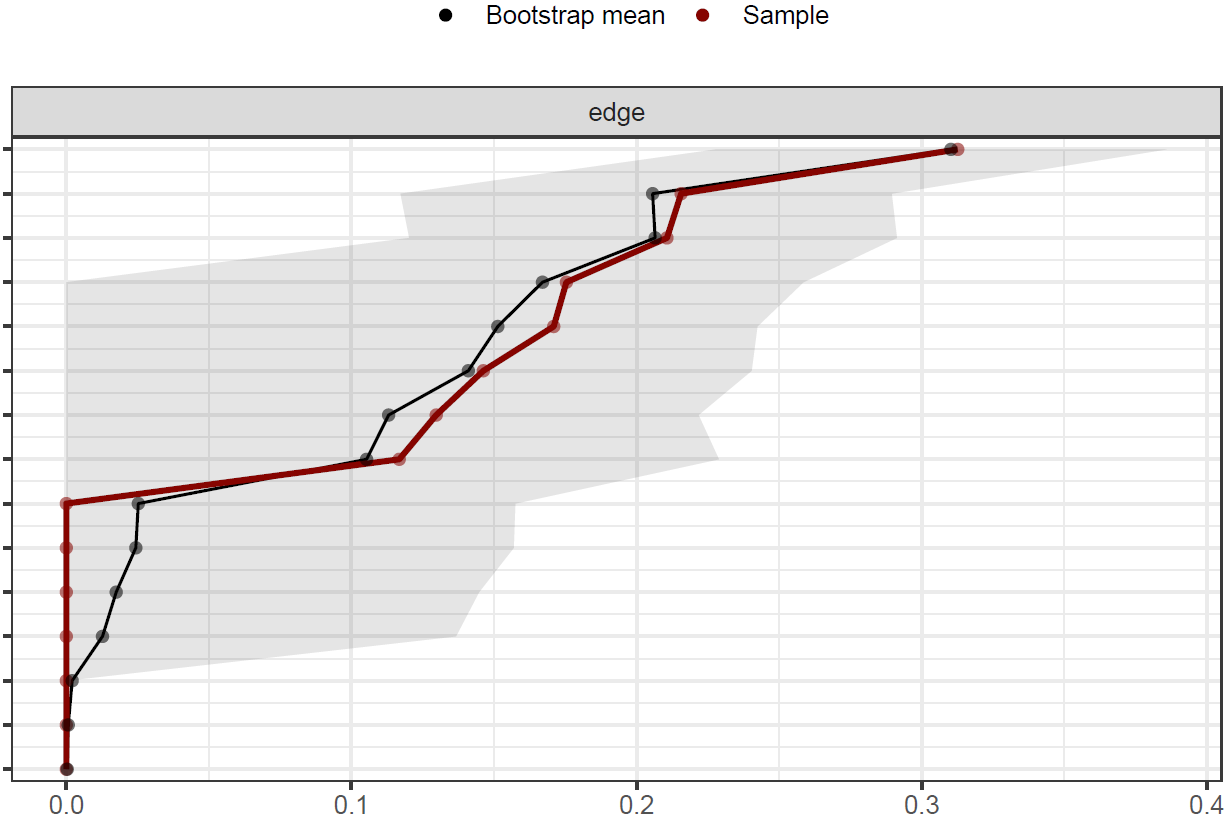


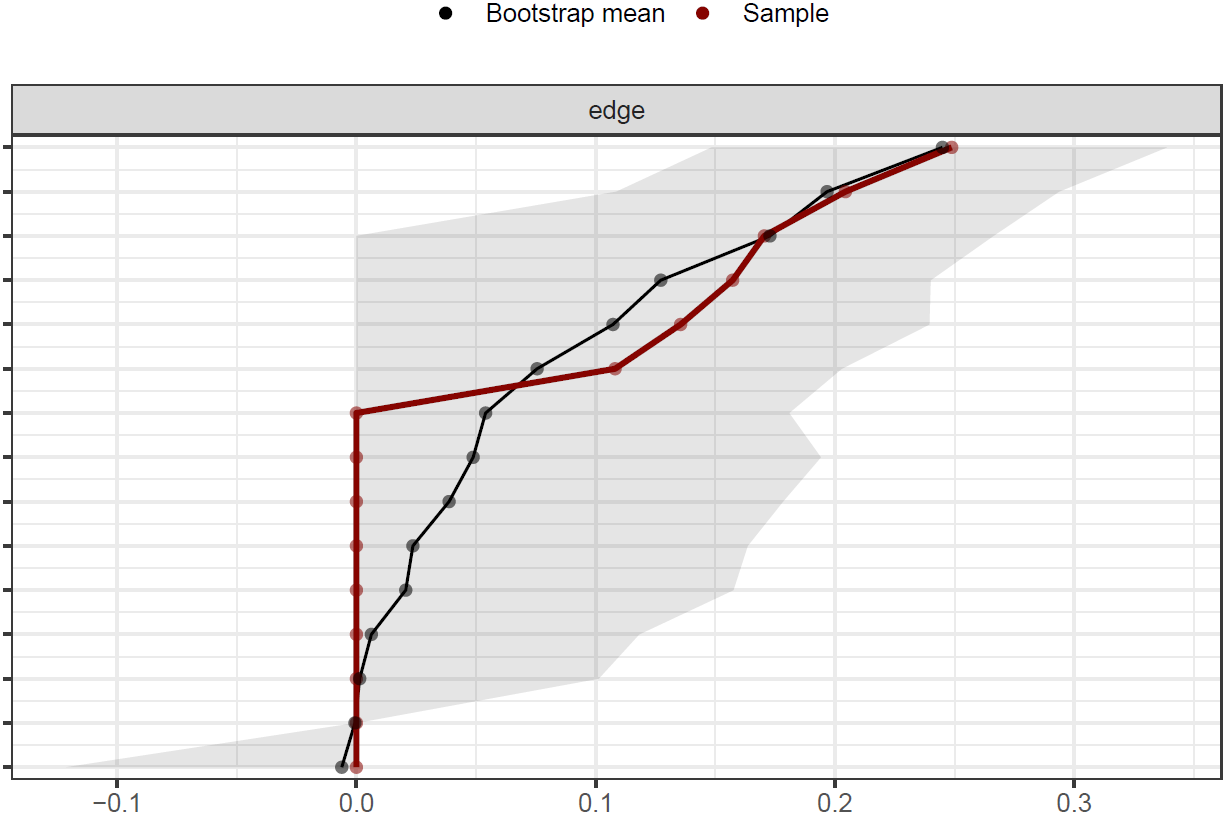


**Fig. S8.** Stability of strength centrality for the PSMU network across participants with low (top) and high (bottom) BSMAS scores.


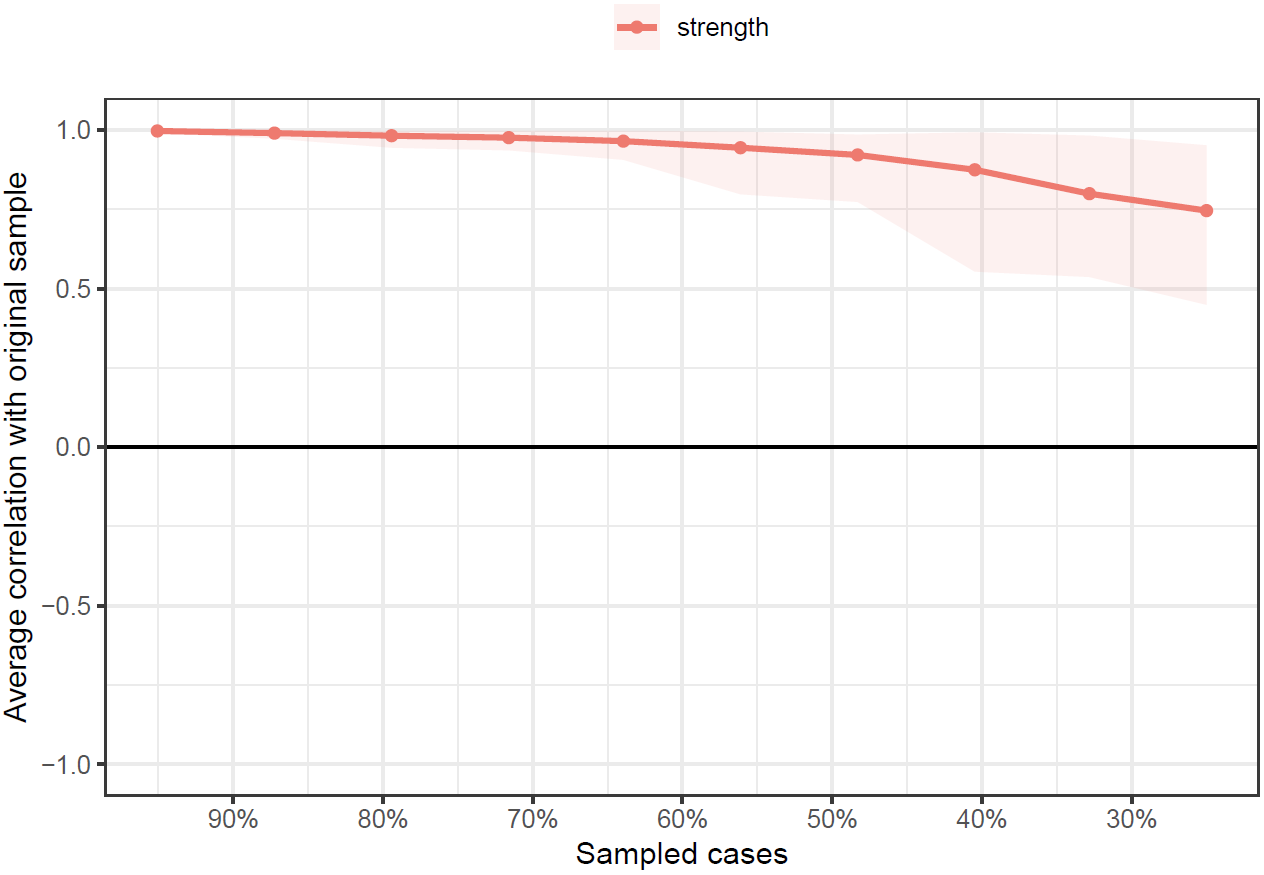


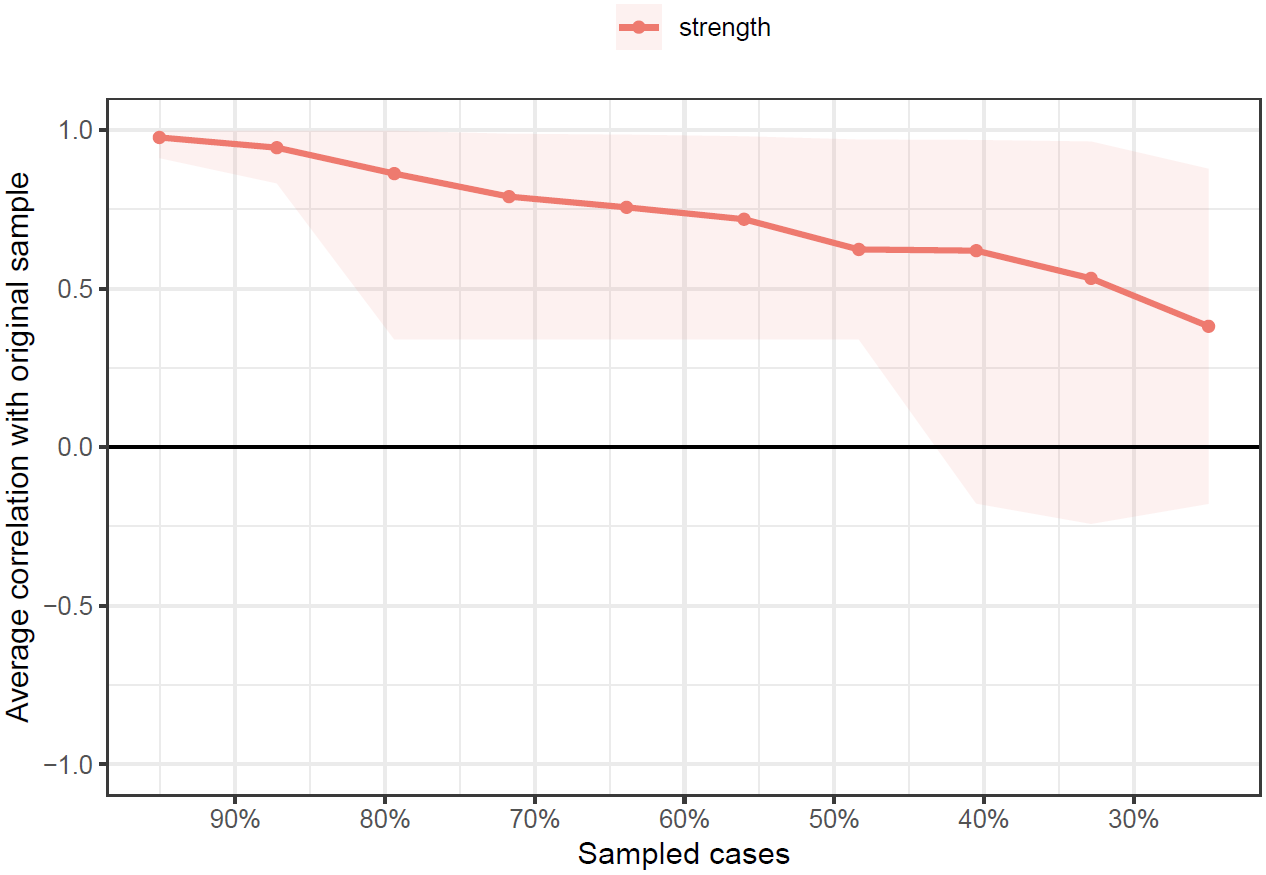

Supplement: Supplementary file 1 [file DataSheet1.doc]
